# A Randomized Open-Labeled Trial of Methotrexate as a Steroid-Sparing Agent for Patients With Generalized Myasthenia Gravis

**DOI:** 10.3389/fimmu.2022.839075

**Published:** 2022-03-18

**Authors:** Li Di, Faxiu Shen, Xinmei Wen, Yan Lu, Wenjia Zhu, Min Wang, Yuwei Da

**Affiliations:** ^1^ Department of Neurology, Xuanwu Hospital, Capital Medical University, Beijing, China; ^2^ Department of Neurology, Beijing Pinggu Hospital, Beijing, China

**Keywords:** generalized myasthenia gravis, methotrexate, steroid-sparing effect, randomized, clinical trial

## Abstract

**Background and Purpose:**

Two clinical trials assessing the steroid-sparing effect of methotrexate (MTX) yielded conflicting results. Our objective was to investigate whether MTX would show a steroid-sparing effect in the treatment of generalized myasthenia gravis (MG) patients who fitted Myasthenia Gravis Foundation of America (MGFA) Class II and Class III.

**Methods:**

We performed an 18-month prospective, randomized, open-labeled trial of prednisone combined with MTX 10 mg orally every week versus prednisone alone in 40 recently diagnosed MG patients of MGFA Class II and Class III between July 2014 and July 2018. The primary endpoint was the prednisone area under the dose–time curve (AUDTC) from months 3 to 18. Secondary endpoints included changes of the Quantitative Myasthenia Gravis Score (QMG), the Myasthenia Gravis Activity of Daily Living Score (MG-ADL), initial time of prednisone reduction, the median prednisone daily dose in each month, adverse events, and treatment failures in each group.

**Results:**

Forty participants were included; among those, 5 individuals withdrew. A total of 35 participants completed 18 months of follow-up (18 in prednisone+MTX, 17 in prednisone group). Combined use of MTX reduced the month 3–18 prednisone AUDTC (prednisone+MTX 5,663.44 ± 1,678.08 mg, prednisone 6,683.94 ± 678.08 mg, *p* = 0.03, 95% confidence interval -1916.01 to -124.98). The initial times of prednisone reduction were 4.34 ± 1.44 months in the prednisone+MTX group and 5.56 ± 2.05 months in the prednisone group (*p* = 0.04, 95% CI -2.41 to -0.03). The median daily prednisone dose was significantly lower in the prednisone+MTX group at month 6 and months 9–18. No significant differences were found in QMG and MG-ADL scores between the two groups. No serious drug-related adverse events were observed in both groups.

**Conclusions:**

This study provides evidence that MTX has the steroid-sparing ability in generalized MG patients of MGFA Class II and Class III.

**Clinical Trial Registration:**

http://www.chictr.org.cn/showproj.aspx?proj=10563 identifier ChiCTR-IPR-15006081.

## Introduction

Corticosteroids are widely recommended as first-line immunotherapy drugs for myasthenia gravis (MG). They exhibit a broad inhibitory effect on the immune system, particularly by T-cell immunosuppression, including interfering with the cooperation of transcription factors responsible for the expression of various proinflammatory cytokines in the cell nucleus, impairing the immunostimulatory function of the monocyte–macrophage lineage, and decreasing the number of circulating T cells (1).

There are numerous systemic side effects associated with long-term corticosteroid therapy, including increased risk of infection, osteoporosis, weight gain, impaired glucose tolerance, diabetes, hypertension, eye diseases (cataract and glaucoma), and neuropsychiatric disturbances (2). Therefore, a non-steroidal immunosuppressive therapy is necessary to reduce the corticosteroid dose to minimize relevant adverse effects or to achieve an earlier steroid-sparing goal.

The steroid-sparing drugs shown to be effective for MG in randomized placebo-controlled studies are azathioprine (AZA) (3) and cyclosporine (4). AZA displayed significant steroid-sparing activity compared with prednisone alone after 15 months of treatment (3). The overall prevalence of hepatotoxicity and myelosuppression was 15.2% and 9.1%, respectively (5). Renal toxicity and potential interactions with other medications make cyclosporine a less favorable treatment choice; 35% of patients discontinue cyclosporine due to cumulative side effects, 10% secondary to slowly progressive nephrotoxicity (4). Steroid-sparing drugs with a more favorable safety profile are much needed. In recent years, two promising candidate drugs, mycophenolate mofetil and tacrolimus, unexpectedly failed to prove efficacy in clinical trials (6–8), although clinical observations suggested effective. Both drugs are expensive and not covered by Chinese health insurance for MG.

There is great interest in looking for alternative steroid-sparing regimens with fewer side effects and a lower price. Emerging treatment choices are biologics with new targets of the immune pathway, like rituximab (8) and eculizumab (9), which are not likely to become the first choice for new diagnosed, immunotherapy-naïve generalized MG patients because of prohibitive costs.

Methotrexate (MTX) is an effective immunosuppressant which has been widely used in rheumatoid arthritis (10) and multiple sclerosis (11). The advantages of MTX include cost-effectiveness, weekly dosing, and moderate side effects. Adverse effects of MTX include cytopenia, infections, liver damage, mucocutaneous toxicity, and hypersensitivity pneumonitis. Serious adverse effects are uncommon in low-dose regimens (5–25 mg/week) (12).

MTX is suggested to work through a number of mechanisms. As a potent inhibitor of dihydrofolate reductase, MTX decreases the *de novo* production of purines and pyrimidines and interferes with DNA synthesis. It can non-specifically prevent T and B cells from proliferating and induce cell apoptosis (13). T cells are highly sensitive to the MTX-induced apoptosis (14). MTX also confers anti-inflammatory properties through the release of adenosine and the inhibition of inflammation mediators (15). Additionally, MTX has also been suggested to modulate the complement system. Decay accelerating factor (DAF) expression at the muscle endplate is an important defense against complement-mediated damage in MG. MTX could increase endogenous DAF level (16). Therefore, the actions of MTX in the treatment of MG may be a complex process of multiple mechanisms acting together rather than a single road.

The efficacy of MTX in MG is still ambiguous. A single-blinded trial proved that MTX had similar efficacy to AZA in generalized MG and an earlier onset of efficacy from 10 months after treatment initiation (17). The other multicenter, randomized, double-blind, placebo-controlled trial of MTX showed that addition of MTX 20 mg for 12 months did not have a significant difference in the month 4–12 prednisone area under the dose–time curve (AUDTC) versus the placebo group (18). The negative results of the latter trial may be because participants were on lower doses of prednisone (median of 20 mg/day) and followed up just 12 months, which may be harder to detect a reduction in prednisone (19–22).

Treatment-naïve patients with moderately severe disease would be the best population to study the benefit of medication (22). However, severe symptoms, as dyspnea and bulb weakness, should be quickly alleviated to prevent MG crisis using a rapid-onset therapeutic regimen, like intravenous immunoglobulin (IVIg) or plasma exchange (23). Generalized MG patients categorized as Myasthenia Gravis Foundation of America (MGFA) Class II or III are common cases in clinic. A low-dose slow-titration regimen of steroid is the rapid-effective and safe therapy for these patients (24). Achieving minimal manifestations soon and reducing prednisone doses are therapeutic goals (25). Therefore, we designed a randomized controlled trial, using a low-dose slow-titration regimen of steroid plus MTX or not, to verify the steroid-sparing effect of MTX for newly diagnosed, immunotherapy-naïve, MGFA Class II and Class III MG patients.

## Methods

### Study Design

We performed a single-center, rater-blinded, randomized controlled trial of MTX in generalized MG Class II and Class III. The trial was approved by the Ethical Committee of Xuanwu Hospital, Capital Medical University, and registered at the Chinese Clinical Trial Registry in 2014 (ChiCTR-IPR-15006081). Written informed consents were signed by all participants. This study followed the Consolidated Standards of Reporting Trials (CONSORT) reporting guideline for randomized clinical trials.

### Participants

All participants were recruited consecutively in the Neurology Department of Xuanwu Hospital between July 2014 and July 2018. The inclusion criteria were defined as follows: (1) eligible participants were between 18 and 80 years of age; (2) patients were newly diagnosed with typical features and either a positive serologic test for acetylcholine receptors or MuSK or a positive edrophonium test and abnormal repetitive nerve stimulation in the case of negative serologic tests; and (3) enrolled patients meet the MGFA classification of Class II or III. Prednisone was prescribed ≥10 mg/day for 1 month prior to enrolling. Patients would be excluded in the following situations: (1) suffered thymoma or had thymectomy within 3 months; (2) previously received MTX or other immunosuppressive agents; (3) had any contraindication to MTX and glucocorticoid use, like tumor, infection, or interstitial lung disease, renal or hepatic insufficiency, fertility planning, women with pregnancy or lactating; (4) presented with comorbid conditions affecting the patients’ strengths, like central nervous system diseases or thyroid diseases.

### Randomization and Blinding

Participants were randomly assigned in a 1:1 ratio to either the prednisone with MTX group or the prednisone alone group using a computer-generated random number sequence provided by the Xuanwu Hospital Clinical Trials Service Unit. Randomization was stratified by severity of disease at time of randomization [quantitative MG (QMG) score ≤10 or >10]. The investigator and all patients were unblinded, but the outcome assessors (FS) remained blinded to group assignment.

### Baseline

All participants had baseline evaluations, including gender, age, body weight, a complete history, disease course, and MGFA classification, and a full laboratory screening including a complete blood and differential count, creatinine, urea and electrolytes, liver transaminases, thyroid hormones, serum glucose and hepatitis B, HIV serological status, and MG antibody subtypes. A chest CT scanning was performed before enrolling to rule out thymoma and interstitial lung disease. The Myasthenia Gravis Activities of Daily Living scale (MG-ADL) and Quantitative Myasthenia Gravis Score (QMG) were evaluated by the assessor (FS) at baseline; pyridostigmine was suspended for at least 10 h prior to performance of the study assessments.

### Interventions

In the prednisone with MTX group, the patients were prescribed with MTX at 7.5 mg/week initially for 2 weeks after enrolling, then increased to 10 mg/week and maintained to the end of the study. The dose selection of MTX was based on the guideline-recommended dose for rheumatoid arthritis in China (26). In the meantime, 5 mg folate was given for 5 days of every week except the day with MTX and the second day. MTX and folate prescriptions were filled at Xuanwu Hospital Pharmacy.

In both groups, pyridostigmine doses ranged between 180 and 360 mg daily depending on symptoms. Oral prednisone was prescribed 10 mg to patients with QMG >10 or 15 mg to patients with QMG ≤10 to avoid temporary exacerbation based on our clinical experience. Prednisone doses were escalated by 5 mg weekly until either 1 mg/kg/day was reached or the patient reached minimal manifestation status (MMS) on a lower dose. The maximum dose of prednisone was maintained for 1 month, while pyridostigmine was gradually discontinued. If MMS was maintained, prednisone would be tapered according to the standardized protocol (**Supplement Table 1**) every month. If MMS could not be maintained, the prednisone daily dose was increased by 5 mg weekly or kept at 1 mg/kg/day until the patient reached MMS again. If patients could not get better, plasmapheresis or IVIg was administered. Vitamin D, calcium supplements, potassium, and acid-inhibitory drugs were prescribed as in standard practice with prednisone.

### Follow-Up

Clinic visits were scheduled monthly for dose adjustment and adverse event reporting. Adverse events were assessed by standard adverse event reporting. MG-ADL and QMG were evaluated by the blinded assessor at 3, 6, 12, and 18 months in the same order. If pyridostigmine was used at that time, it would be suspended for at least 10 h prior to performance of the study assessments.

The following laboratory tests were monitored weekly for the first 8 weeks and monthly thereafter: complete blood counts, serum aspartate aminotransferase (AST), alanine aminotransferase (ALT), gamma glutamyltransferase (GGT), creatinine, urea nitrogen, and random glucose level.

### Outcomes

The primary endpoint was 15-month (months 3–18) prednisone area under the dose–time curve (AUDTC). Prednisone AUDTC measured total prednisone dose. A difference between the prednisone with MTX group and the prednisone alone group could prove a steroid-sparing effect of MTX. The secondary endpoints included the changes of MG-ADL and QMG from baseline, initial time of prednisone reduction, the median prednisone daily dose in each month, adverse events, and treatment failures in each group. Treatment failure was defined as uncontrolled MG symptoms in 1 year or more than one relapse requiring increased prednisone dose.

### Sample Size

The sample size was decided based on a prior study in MG (27). We assumed a mean AUDTC/SD ratio of 3 for the prednisone-alone group and equal variances between treatment groups. A 33% reduction in the mean AUDTC/SD in the prednisone with methotrexate group was considered clinically relevant. The sample size of 40 participants (20 patients in the prednisone with methotrexate group and 20 patients in the prednisone alone group) provided a power of 80% to detect a 33% difference in the mean AUDTC/SD with a two-sided α level of 5%, allowing for a 20% dropout rate.

### Statistical Analysis

Efficacy analyses were done in the modified intention-to-treat population, including all randomly assigned patients who had a valid baseline assessment and at least one post-baseline assessment. Safety analyses were done in all patients who received at least one dose of prednisone or MTX. Patients were discontinued from treatment if they received rescue therapy (plasma exchange or IVIg) or developed a serious adverse event that could jeopardize their safety. After discontinuation, patients who did not withdraw consent were followed up for safety and disease severity assessments through the rest of the trial.

Normally distributed data were presented as mean and standard deviation (SD). Balanced randomization was tested using an independent-sample *t* test if the normality assumption was satisfied. The non-normally distributed data were presented as median and interquartile ranges (IQR), and the rank-sum test was used. The composition of gender and MGFA Classifications were tested by the chi-square test between the two groups. All statistical tests were two-sided. A *p* value of <0.05 was considered statistically significant. Analysis was performed using SPSS 17.0.

## Results

**Figure 1:**
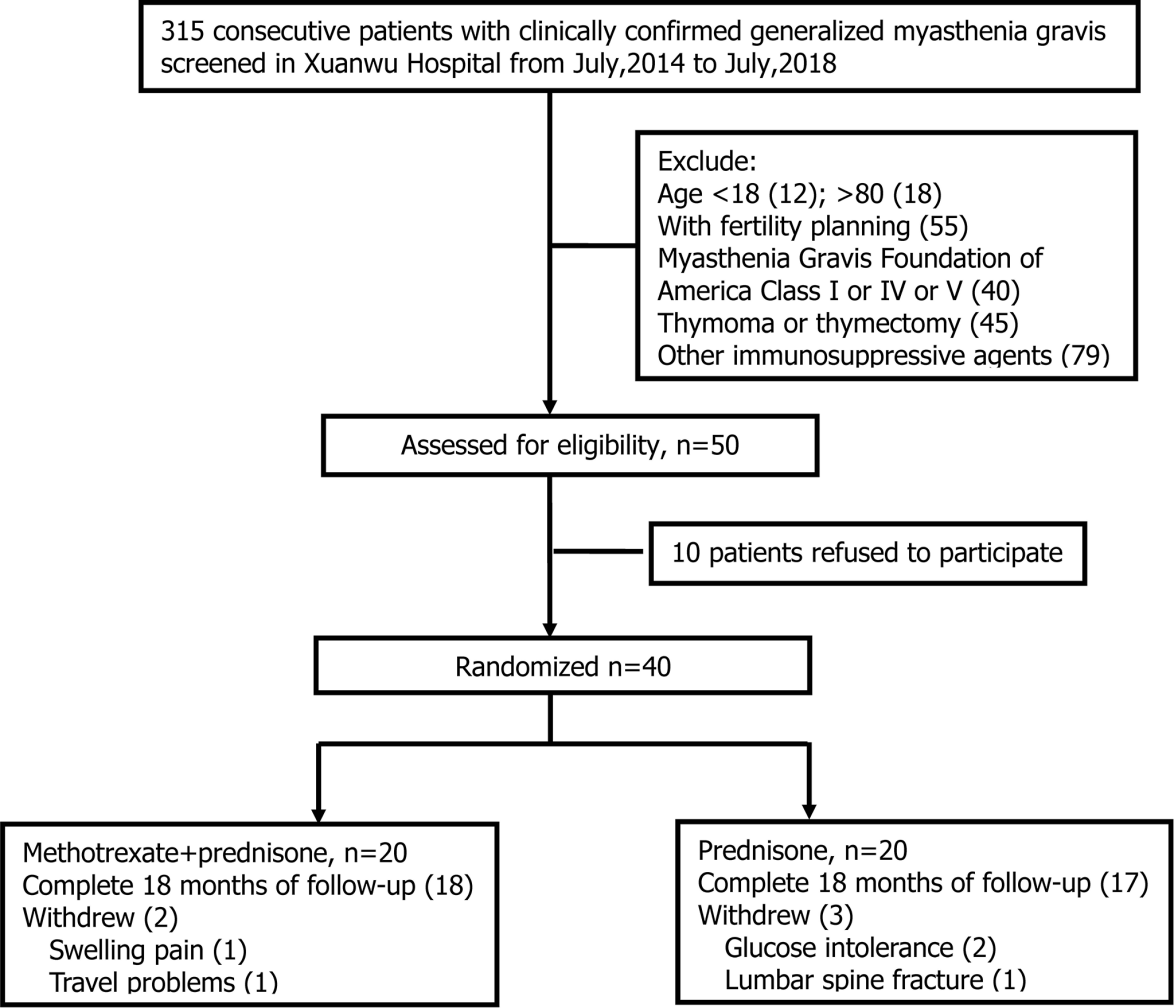
A flowchart for subject selection and reasons for withdrawal.

Three hundred and fifteen patients were clinically diagnosed with MG between July 2014 and July 2018 in our center. Two hundred and sixty-five patients did not meet the inclusion criteria for the following reasons: 1) 30 patients were ineligible for age; 2) 55 patients had fertility planning; 3) 40 patients were MGFA class I or IV or V; 4) 45 patients had thymoma or thymectomy within 3 months; 5) 79 patients chose to use other immunosuppressive agents (tacrolimus, mycophenolate mofetil, etc.); and 6) 16 patients had contraindications to MTX or glucocorticoid (elevated liver enzyme or severe osteoporosis). Of the 50 eligible patients, 10 declined to participate after screening, and 40 were randomized to prednisone with the MTX group or to the prednisone group in a 1:1 ratio (**Figure 1**). Five participants withdrew in the first month of observation: 1 due to swelling pain in interphalangeal joints, 1 due to travel problems in the prednisone with MTX group, 2 due to glucose intolerance, and 1 due to lumbar spine fracture in the prednisone group. Finally, 35 patients completed 18 months of follow-up, including 18 in the prednisone with MTX group and 17 in the prednisone group. A total of 35 patients were included in the analysis. All outcomes of 35 participants were intact.

### Baseline Characteristics

**Figure 2:**
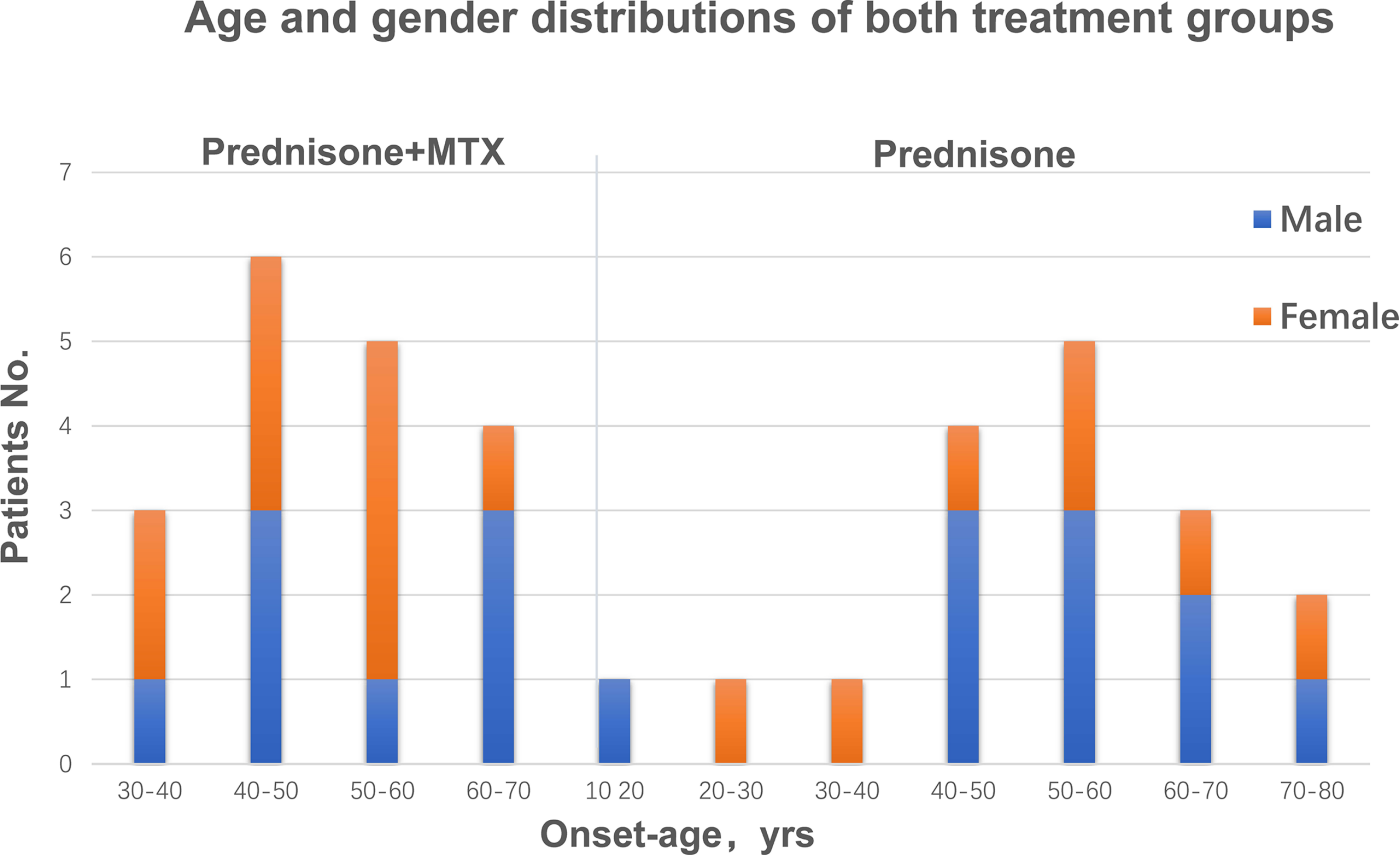
Age and gender distributions of both treatment groups.

Both groups were comparable in regard to gender, age, and body weight. There were 8 males and 10 females in the prednisone+MTX group and 10 males and 7 females in the prednisone-alone group (**Table 1**). According to the onset age, there were 9 early-onset patients and 9 late-onset patients in the prednisone+MTX group, while 7 early-onset patient and 10 late-onset patients in the prednisone-alone group (**Table 1** and **Figure 2**). The onset age of early-onset patients ranged from 18 to 49 years, and that of late-onset patients ranged from 50 to 72 years (**Figure 2**). All participants had 2–3 months of disease duration (**Table 1**). 67% and 59% of participants were MGFA Class III in the prednisone+MTX and prednisone-alone groups, respectively. 50% of early-onset MG patients were MGFA Class III, and 73.7% of late-onset MG patients were MGFA Class III. More bulbar symptoms and neck weakness occurred in late-onset MG patients (78.9%) than early-onset patients (56.3%). 89% cases (16/18) in the prednisone+MTX group and 76% cases (13/17) in the prednisone group were AChR-Ab positive. No positive MuSK-Ab was found in all cases. The above two antibody titers were assessed by the radioimmunoassay method. We did not test for the LRP4 antibody. The diagnoses of seronegative cases were based on typical clinical features, positive edrophonium test, and abnormal repetitive nerve stimulation study. QMG and MG-ADL scores were balanced between two treatment groups at baseline. Initial prednisone daily doses were 25.27 ± 6.30 mg in the prednisone+MTX group and 22.06 ± 6.86 mg in the prednisone group (*p* = 0.16). Concomitant diseases were similar in both groups, and all occur in the late-onset patients.

**Table 1:** Baseline characteristics of both groups.

	MTX + PRED	PRED	*p* value
Patients, n (male/female)	18 (8/10)	17 (10/7)	0.61*^a^*
Age (year), mean ± SD	49.22 ± 10.94	50.47 ± 14.89	0.78*^b^*
Early onset, n (male/female)	9 (4/5)	7 (4/3)	0.51*^a^*
Late onset, n (male/female)	9 (4/5)	10 (6/4)	0.67*^a^*
Weight (kg), mean ± SD	67.91 ± 12.86	67.20 ± 8.92	0.85*^b^*
Disease course (month), mean ± SD	2.36 ± 1.56	2.55 ± 1.59	0.70*^b^*
MGFA classification, n (%)			
II	6 (33%)	7 (41%)	0.70*^a^*
III	12 (67%)	10 (59%)	0.56*^a^*
AChR-Ab positive, (n)	16 (89%)	13 (76%)	0.33*^a^*
Musk-Ab positive, (n)	0	0	
Seronegative, (n)	2 (11%)	4 (24%)	
QMG, mean ± SD	10.83 ± 5.15	11.11 ± 4.83	0.87*^b^*
MG-ADL, mean ± SD	8.61 ± 4.88	7.47 ± 2.94	0.41*^b^*
Initial prednisone daily dose (mg), mean ± SD	25.27 ± 6.30	22.06 ± 6.86	0.16*^b^*
Concomitant disease, n			
Hypertension	2	2	
Diabetes	1	0	
Rheumatoid arthritis	1	1	

MTX, methotrexate; PRED, prednisone; MGFA, Myasthenia Gravis Foundation of America; QMG, Quantitative Myasthenia Gravis; ADL, Activities of Daily Living Score; SD, standard deviation.

^a^The χ^2^ test was used for group comparison if the expected cell counts were larger than 5; otherwise, the Fisher exact test was used.

^b^Independent-samples t test was used for group comparison if the normality assumption was satisfied; otherwise, the Wilcoxon rank-sum test was used.

### Outcomes and Measures

There was a significant difference in 3–18 months of prednisone AUDTC between the two groups (prednisone+MTX 5,663.44 ± 1,678.08 mg, prednisone 6,683.94 ± 1,041.96 mg, *p* = 0.03, 95% confidence interval [CI]-1916.01 to -124.98) (**Table 2**). No significant difference in early- vs. late-onset subgroups was found, although the mean prednisone AUDTC seemed lower in the early-onset subgroup with prednisone+MTX treatment (**Table 2**). The only significant difference was found in the male early-onset vs. late-onset subgroup. The prednisone AUDTC of male early-onset patients was 4,859.50 ± 1,145.88 mg in the prednisone+MTX group and 6,818.00 ± 366.09 mg in the prednisone alone group (*p* = 0.017, 95% confidence interval -3,430.25 to -486.75). No difference was found in the male late-onset subgroup and female patients between two treatment groups.

**Table 2:** Outcome measures.

Prednisone AUDTC, mean ± SD	MTX + PRED	PRED	95% CI	*p* value
3–18 months AUDTC	5,663.44 ± 1,678.08	6,683.94 ± 1,041.96	-1,916.01,-124.98	**0.03**
Early-onset subgroup	5,393.78 ± 1,312.71	6,695.14 ± 1,258.98	-2,695.64, 92.91	0.065
Male	4,859.50 ± 1,145.88	6,818.00 ± 366.09	-3,430.25, -486.75	**0.017**
Female	5,821.20 ± 1,395.64	6,531.33 ± 2,117.46	-3,696.62, 2,276.35	0.582
Late-onset subgroup	6,022.00 ± 1,598.41	6,675.90 ± 1,450.94	-2,129.43, 821.63	0.363
Male	5,444.75 ± 2,172.59	6,663.33 ± 1,712.99	-4,044.42,1,607.26	0.349
Female	6,483.80 ± 984.83	6,694.75 ± 1,193.45	-1,922.80,1,500.90	0.779
Prednisone reducing time (month)	4.34 ± 1.44	5.56 ± 2.05	-2.41, -0.03	**0.04**
QMG at baseline, mean ± SD	10.83 ± 5.15	11.11 ± 4.83		0.87
ΔQMG				
Baseline–3 months	4.17 ± 4.08	6.47 ± 3.47	-4.91,0.31	0.08
Baseline–6 months	6.83 ± 5.11	8.47 ± 5.54	-5.30,2.02	0.37
Baseline–12 months	9.00 ± 5.40	8.82 ± 6.02	-3.75,4.11	0.93
Baseline–18 months	9.78 ± 5.30	9.82 ± 5.07	-3.61,3.52	0.98
MG-ADL at baseline, mean ± SD	8.61 ± 4.88	7.47 ± 2.94		0.41
ΔMG-ADL				
Baseline–3 months	5.67 ± 4.13	5.76 ± 3.44	-2.72,2.52	0.94
Baseline–6 months	6.72 ± 3.95	6.24 ± 3.61	-2.12,3.10	0.71
Baseline–12 months	8.17 ± 4.63	6.76 ± 3.44	-1.42,4.22	0.32
Baseline–18 months	8.56 ± 4.72	7.00 ± 3.26	-1.25,4.36	0.27

AUDTC, area under the dose time curve; CI, confidence interval; SD, standard deviation; Δ, mean difference; ADL, Activities of Daily Living Score; QMG, quantitative myasthenia gravis.

P value which is less than 0.05 were showed in bold.

All patients discontinued pyridostigmine within 3 months. The initial time of prednisone reduction in the prednisone+MTX group was 4.34 ± 1.44 months, and that in the prednisone group was 5.56 ± 2.05 months (*p* = 0.04, 95% CI -2.41 to -0.03). The assessments of QMG and MG-ADL scores in months 3, 6, 12, and 18 did not show significant differences between two treatment groups (**Table 2**). Bulbar weakness recovered sooner than other symptoms, while ocular symptoms were persistent in both treatment groups. Tears and photophobia were the most common complains in the prednisone group, especially in late-onset patients.

**Figure 3:**
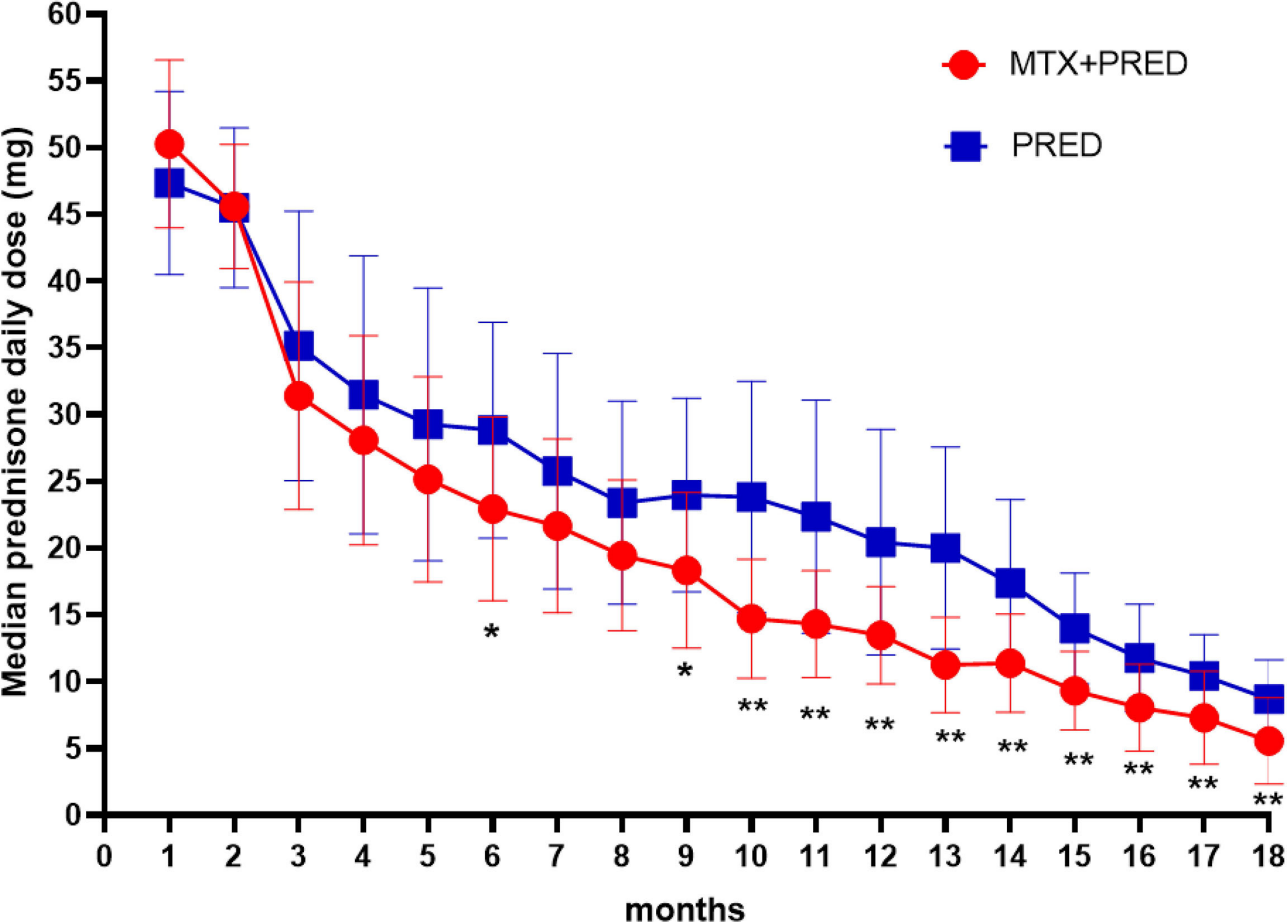
Median daily prednisone dose by month of two groups. MTX, methotrexate; PRED, prednisone. Error bars: standard deviation. *p < 0.05, **p < 0.0.

The decrease of median prednisone daily dose in the prednisone+MTX group started at month 6 (prednisone+MTX 22.92 ± 6.87 mg, prednisone 28.82 ± 8.11 mg, *p* = 0.026, 95% CI -11.07 to -0.75) and became more significant from month 9 (prednisone+MTX 18.33 ± 5.82 mg, prednisone 23.97 ± 7.24 mg, *p* = 0.016, 95% CI -10.14 to -1.13) to month 18 (*p* < 0.01 in months 10–18) (**Figure 3**, **Table 3**). When prednisone dose was decreased to 15–25 mg/day, 1 participant in the prednisone with MTX group and 6 in the prednisone group suffered symptom worsening and prednisone dose increase. One participant in the prednisone with MTX group discontinued prednisone from month 12 to the end of the trial. There were 8 participants (44%) in the prednisone with MTX group and 3 participants (18%) in the prednisone group with median prednisone daily dose ≤5 mg at month 18.

**Table 3:** Median daily prednisone dose by month of the two groups.

	The median daily dose of prednisone (mg, mean ± SD)			
Month	PRED + MTX	PRED	95% CI	*p value*
1	50.28 ± 6.29	47.35 ± 6.86	-5.89, 2.38	0.395
2	45.60 ± 4.67	45.49 ± 5.98	-3.38, 2.32	0.707
3	31.39 ± 8.54	35.15 ± 10.10	-10.18, 2.66	0.242
4	28.06 ± 7.84	31.47 ± 10.42	-9.73, 2.90	0.279
5	25.14 ± 7.69	29.26 ± 10.22	-10.32, 2.07	0.185
6	22.92 ± 6.87	28.82 ± 8.11	-11.07, -0.75	**0.026**
7	21.67 ± 6.53	25.74 ± 8.83	-9.39, 1.25	0.129
8	19.44 ± 5.66	23.38 ± 7.60	-8.53, 0.65	0.090
9	18.33 ± 5.82	23.97 ± 7.24	-10.14, -1.13	**0.016**
10	14.72 ± 4.45	23.82 ± 8.67	-13.95, -4.25	**0.001**
11	14.31 ± 4.00	22.35 ± 8.73	-12.85, -3.24	**0.002**
12	13.47 ± 3.65	20.44 ± 8.44	-11.58, -2.36	**0.005**
13	11.25 ± 3.56	20.00 ± 7.55	-12.92, -4.58	**0.000**
14	11.39 ± 3.66	17.35 ± 6.28	-9.57, -2.36	**0.002**
15	9.31 ± 2.95	13.97 ± 4.15	-7.13, -2.20	**0.001**
16	8.06 ± 3.27	11.76 ± 4.03	-6.23, -1.19	**0.005**
17	7.29 ± 3.49	10.44 ± 3.09	-5.42, -0.88	**0.008**
18	5.56 ± 3.24	8.68 ± 2.95	-5.26, -0.99	**0.005**

PRED, prednisone; MTX, methotrexate; CI, confidence interval; SD, standard deviation.

P value which is less than 0.05 were showed in bold.

No treatment failure happened in each group. No participant had uncontrolled MG symptoms in 1 year or twice relapses or severe situation requiring hospitalization for IVIg or plasmapheresis.

### Adverse Events and Treatment Failure

There were a total of 37 adverse events reported (17 events in the prednisone+MTX group, 20 events in the prednisone group) (**Table 4**). Four patients withdrew due to the adverse events: one due to the swelling pain in hand joints in the prednisone+MTX group, 2 due to glucose intolerance, and 1 due to lumbar spine fracture in the prednisone group. Other adverse events did not result in any participant quitting the study. Weight gain was the most common adverse event. There were similar cases with insomnia, glucose increase, skin infections, and leukocytosis in both groups. Two patients had elevated liver function tests (<3× upper limit of normal), and one had loss of appetite in the MTX group. Two patients had acid reflux and regurgitation, and two patients suffered osteoporosis without fracture in the prednisone group. Abnormal liver function and folliculitis mostly occurred in early-onset patients, while glucose increase and osteoporosis were the common adverse events in late-onset patients. There were no MTX-related serious adverse events.

**Table 4:** Adverse events.

Adverse events	Total events in prednisone + MTX group (n = 17)	Total events in prednisone group (n = 20)
Pain	1	0
Bone fracture	0	1
Weight gain	6	6
Insomnia	1	2
Glucose increase	2	3
Onychomycosis	1	1
Folliculitis	1	1
Leukocytosis	2	2
Abnormal liver function	2	0
Gastrointestinal symptoms	1	2
Osteoporosis	0	2

## Discussion

Two trials assessing the steroid-sparing effect of methotrexate generated conflicting results (17, 18); our study added a favorable evidence for MTX in the treatment of mild to moderate generalized MG. The month 3–18 prednisone AUDTC in the prednisone with MTX group was significantly lower than that in the prednisone group. Fewer patients suffered symptom worsening and prednisone dose increase, and more patients took the median prednisone daily dose of ≤5 mg at month 18 in the prednisone with MTX group. The assessment of QMG and MG-ADL scores at months 3, 6,12, and 18 showed significant improvements in both groups, and no serious adverse event happened in patients with MTX. These revealed that MTX had steroid-sparing benefit and was well-tolerated in the generalized MG patients of MGFA Class II and Class III.

The trial of methotrexate versus placebo for generalized MG showed that MTX did not reduce the month 4–12 prednisone AUDTC (18), which had a number of major differences from our study. (1) Our study included younger participants with short MG symptom durations (2–3 months) and in the phase of escalating prednisone dose. These patients may be more easily observed to have the effect of immunotherapy. (2) Although there were similar QMG scores at enrolling in both studies, the changes of mean 12-month QMG were more prominent in our study than those in the methotrexate versus placebo trial (**Table 5**), which showed that our patients had more obvious symptomatic improvement. (3) The primary endpoint of our study was the prednisone AUDTC from months 3 to 18, whereas the methotrexate versus placebo trial was prednisone AUDTC from months 4 to 12, which may be too short to manifest the difference (20).

**Table 5:** The comparison of three trials of MTX for generalized MG.

	MTX vs. Azathioprine	MTX vs. Placebo	The present study
Blind method	Single-blind	Double-blind	Single-blind
Patients, n	15 vs. 16	25 vs. 25	18 vs. 17
Age, year	42.7 vs. 47.9	66.5 vs. 68.6	49.2 vs. 50.5
Observation period, month	24	12	18
MGFA classification at baseline	Class 2 19%	Class 2 86%	Class 2 37%
	Class 3 35%	Class 3 14%	Class 3 63%
	Class 4 35%		
	Class 5 11%		
QMG at baseline	19.5 vs. 20	10.5 vs. 10.4	10.83 vs. 11.11
MG-ADL at baseline	7.5 vs. 6.0	4.8 vs. 4.1	8.61 vs. 7.47
Disease course (month)	10.3 vs. 7.5	NM	2.36 vs. 2.55
AChR-Ab-positive (%)	61%	100%	83%
Initial prednisone dose (mg)	NM	20	25.27 vs. 22.06
MTX dose (mg/w)	17.5	20	10
QMG change (baseline–the end)	14 vs. 11.5	-1.4 vs. 0.3	10 vs. 9.82
MG-ADL change (baseline–the end)	6.0 vs. 7.5	-1.2 vs. 0.26	8.44 vs. 7.0

MTX, methotrexate; MGFA, Myasthenia Gravis Foundation of America; QMG, quantitative myasthenia gravis; ADL, Activities of Daily Living Score; NM, not mentioned.

We calculated 3–18-month prednisone AUDTC as a primary outcome, because the effect of MTX would not occur immediately and month 3–18 prednisone AUDTC closely mimicked prednisone dosing decrement processes based on MG symptoms. The maximum doses were all reached by month 2 following the prednisone-escalating rule (5 mg weekly until either 1 mg/kg/day was reached or a lower dose in those who reached MMS). Month 3–18 prednisone AUDTC could demonstrate the steroid-sparing effect of MTX in the MG maintenance treatment period.

It is not clear when MTX shows a steroid-sparing effect in the treatment of MG. The methotrexate versus AZA trial showed that MTX had an onset of steroid-sparing efficacy from 10 to 12 months. It was concluded from the significantly lower average daily prednisone doses at months 10 and 12 in the MTX group (17). Our study revealed that the initial time of prednisone reduction was earlier in the prednisone+MTX group (4.34 ± 1.44 months) than the prednisone group (5.56 ± 2.05 months), indicating that addition of MTX could help MG patients achieve MMS 1 month earlier. It suggested that MTX might also have an effect on the induction therapy period. The difference of the median daily prednisone dose between both groups started at month 6 and became more significant in months 9–18, which revealed that MTX’s steroid-sparing effect might start earlier. The reason is probably that we compared prednisone+MTX to prednisone alone, while they compared prednisone+MTX to prednisone+AZA (17). Another reason may be that our participants were in the milder MGFA classifications, lower QMG scores, and shorter MG durations than those in the trial of methotrexate versus AZA (**Table 5**), which may imply that MTX might be more suitable for mild cases with short duration. Baseline and improvements of MG-ADL at the end of our trial were higher than those in the trial of methotrexate versus AZA (**Table 5**), probably because patients were new-onset and thus more likely to feel worse at baseline.

It is worth noting that there was a marked increase of the accumulative monthly prednisone dose from month 9 (350 mg/month) to month 10 (450 mg/month) in the placebo group of the methotrexate versus placebo trial [see the Supplemental Figure e-1 of Ref (18)]. Likewise, in our study, there was also an increase of the prednisone median daily dose at months 9–10 in the prednisone-alone group. Six participants in the prednisone group versus 1 participant in the prednisone+MTX group needed to increase prednisone doses when prednisone reduced to 15–25 mg/day. These indicate that we should pay more attention to fluctuations of MG symptoms in this period. Addition of MTX may reduce the occurrence of fluctuations when prednisone was at a relatively low dose.

We set the MTX dose according to the Chinese guideline for treatment of rheumatoid arthritis (26). The efficacy of MTX 10 mg/week had been confirmed in RA patients. A tight control treatment approach revealed that MTX 10 mg/week might be a good choice for most RA patients and is frequently already the optimal dose (28). A systematic review suggested that for early RA patients who start on MTX as monotherapy or in combination with glucocorticoids, a higher dose of MTX was not associated with better clinical outcomes (29). A randomized, double-blind, parallel-armed study showed that no differences in disease activity, radiographic progression, or functional ability response were found between MTX dosages of 10 or 20 mg/week in combination with adalimumab (30). Our study showed that MTX 10 mg/week had the therapeutic effect in generalized MG subjects and fewer side effects than two previous studies (17, 18). The incident rate of non-special pain and gastrointestinal side effect in the MTX 20 mg/week trial were 56% and 60%, respectively (18). In our study, only 5.6% of participants reported joint pain, 5.6% of participants reported loss of appetite, and 11.2% of participants had abnormal liver function in the MTX group. Low-dose MTX was the primary reason for fewer adverse events.

There are some limitations in this trial. It was a small-sized, open-labeled trial without placebo, which had inevitable potential bias on both clinician and participants. There was the least prednisone AUDTC in the early-onset male subgroup with prednisone+MTX, but we could not come to the conclusion that the prednisone+MTX therapeutic strategy would be more suitable for early-onset male patients due to the small size of subgroup in this trial, which needs to be validated in a large-scale population. The conclusion was drawn from newly diagnosed, immunotherapy-naïve, MGFA Class II and Class III MG patients, indicating that it may not be suitable for all MG patients.

## Conclusions

This prospective, randomized trial provides evidence that MTX has steroid-sparing potential in generalized MG patients with MGFA Class II and Class III. Methotrexate with its advantages of once weekly dosing, mild side effects, and affordable price is expected to be a steroid-sparing agent for mild to moderate generalized MG patients, especially in financially constrained health systems.

## Data Availability Statement

The raw data supporting the conclusions of this article will be made available by the authors, without undue reservation.

## Ethics Statement

The studies involving human participants were reviewed and approved by the Institutional Ethical Review Board of Xuanwu Hospital, Capital Medical University. The patients/participants provided their written informed consent to participate in this study.

## Author Contributions

LD and YD designed the study. LD and FS collected all the clinical data, carried out the data processing and statistical analysis, and drafted the manuscript. YL, WZ, and MW cared for the enrolled patients. XW reviewed the literature and revised the manuscript. YD was involved in the study concept and design, critical revision of the manuscript, procurement of funding, study supervision, and final approval of the version to be published. All authors contributed to the article and approved the submitted version.

## Funding

The study was funded by the Clinical Cohort Study of Myasthenia Gravis, National Key R&D Program of China, Precision Medicine Project (No. 2017YFC0907700); The National Natural Science Foundation of China (82101470, 82001352, 81801255); Capital Health Development Research Project (2018-4-2015); and Beijing Hospitals Authority Youth Program (QML20190803).

## Conflict of Interest

The authors declare that the research was conducted in the absence of any commercial or financial relationships that could be construed as a potential conflict of interest.

## Publisher’s Note

All claims expressed in this article are solely those of the authors and do not necessarily represent those of their affiliated organizations, or those of the publisher, the editors and the reviewers. Any product that may be evaluated in this article, or claim that may be made by its manufacturer, is not guaranteed or endorsed by the publisher.

## References

[1] Sathasivam S. Steroids and Immunosuppressant Drugs in Myasthenia Gravis. Nat Clin Pract Neurol (2008) 4:317–27. doi: 10.1038/ncpneuro0810 18493241

[2] Gilhus NE Tzartos S Evoli A Palace J Burns TM Verschuuren J. Myasthenia Gravis. Nat Rev Dis Primers (2019) 5:30. doi: 10.1038/s41572-019-0079-y 31048702

[3] Palace J Newsom-Davis J Lecky B. A Randomized Double-Blind Trial of Prednisolone Alone or With Azathioprine in Myasthenia Gravis. Myasthenia Gravis Study Group. Neurology (1998) 50(6):1778–83. doi: 10.1212/wnl.50.6.1778 9633727

[4] Tindall RS Phillips JT Rollins JA Wells L Hall K. A Clinical Therapeutic Trial of Cyclosporine in Myasthenia Gravis. Ann N Y Acad Sci (1993) 681:539–51. doi: 10.1111/j.1749-6632.1993.tb22937.x 8357194

[5] Jack KL Koopman WJ Hulley D. Nicolle MW. A Review of Azathioprine-Associated Hepatotoxicity and Myelosuppression in Myasthenia Gravis. J Clin Neuromuscul Dis (2016) 18(1):12–20. doi: 10.1097/CND.0000000000000133 27552384

[6] Sanders DB Hart IK Mantegazza R Shukla SS Siddiqi ZA De Baets MH. An International, Phase III, Randomized Trial of Mycophenolate Mofetil in Myasthenia Gravis. Neurology (2008) 71(6):400–6. doi: 10.1212/01.wnl.0000312374.95186.cc 18434638

[7] Yoshikawa H Kiuchi T Saida T Takamori M. Randomised, Double-Blind, Placebo-Controlled Study of Tacrolimus in Myasthenia Gravis. J Neurol Neurosurg Psychiatry (2011) 82(9):970–7. doi: 10.1136/jnnp-2011-300148 21784757

[8] Zhou L Liu W Li W Li H Zhang X Shang H. Tacrolimus in the Treatment of Myasthenia Gravis in Patients With an Inadequate Response to Glucocorticoid Therapy: Randomized, Double-Blind, Placebo-Controlled Study Conducted in China. Ther Adv Neurol Disord (2017) 10(9):315–25. doi: 10.1177/1756285617721092 PMC5557184 28861121

[9] Howard JJ Utsugisawa K Benatar M Murai H Barohn RJ Illa I. Safety and Efficacy of Eculizumab in Anti-Acetylcholine Receptor Antibody-Positive Refractory Generalised Myasthenia Gravis (REGAIN): A Phase 3, Randomised, Double-Blind, Placebo-Controlled, Multicentre Study. Lancet Neurol (2017) 16(12):976–86. doi: 10.1016/S1474-4422(17)30369-1 29066163

[10] Jeurissen ME Boerbooms AM van de Putte LB Doesburg WH Mulder J Rasker JJ. Methotrexate Versus Azathioprine in the Treatment of Rheumatoid Arthritis. A Forty-Eight-Week Randomized, Double-Blind Trial. Arthritis Rheum (1991) 34(8):961–72. doi: 10.1002/art.1780340805 1859490

[11] Goodkin DE Rudick RA VanderBrug MS Daughtry MM Schwetz KM Fischer J. Low-Dose (7.5 Mg) Oral Methotrexate Reduces the Rate of Progression in Chronic Progressive Multiple Sclerosis. Ann Neurol (1995) 37(1):30–40. doi: 10.1002/ana.410370108 7818255

[12] Lucas CJ Dimmitt SB Martin JH. Optimising Low-Dose Methotrexate for Rheumatoid Arthritis-A Review. Br J Clin Pharmacol (2019) 85:2228–34. doi: 10.1111/bcp.14057 PMC6783593 31276602

[13] Chan ES Cronstein BN. Methotrexate–How Does it Really Work? Nat Rev Rheumatol (2010) 6:175–8. doi: 10.1038/nrrheum.2010.5 20197777

[14] Herman S Zurgil N Deutsch M. Low Dose Methotrexate Induces Apoptosis With Reactive Oxygen Species Involvement in T Lymphocytic Cell Lines to a Greater Extent Than in Monocytic Lines. Inflamm Res (2005) 54:273–80. doi: 10.1007/s00011-005-1355-8 16134056

[15] Kozminski P Halik PK Chesori R Gniazdowska E. Overview of Dual-Acting Drug Methotrexate in Different Neurological Diseases, Autoimmune Pathologies and Cancers. Int J Mol Sci (2020) 21(10):14. doi: 10.3390/ijms21103483 PMC7279024 32423175

[16] Auret J Abrahams A Prince S Heckmann JM. The Effects of Prednisone and Steroid-Sparing Agents on Decay Accelerating Factor (CD55) Expression: Implications in Myasthenia Gravis. Neuromuscul Disord (2014) 24:499–508. doi: 10.1016/j.nmd.2014.02.010 24703255

[17] Heckmann JM Rawoot A Bateman K Renison R Badri M. A Single-Blinded Trial of Methotrexate Versus Azathioprine as Steroid-Sparing Agents in Generalized Myasthenia Gravis. BMC Neurol (2011) 11:97. doi: 10.1186/1471-2377-11-97 21819556 PMC3170595

[18] Pasnoor M He J Herbelin L Burns TM Nations S Bril V. A Randomized Controlled Trial of Methotrexate for Patients With Generalized Myasthenia Gravis. Neurology (2016) 87(1):57–64. doi: 10.1212/WNL.0000000000002795 27306628 PMC4932232

[19] Drachman DB. Comment: Methotrexate for Patients With Generalized Myasthenia Gravis. Neurology (2016) 87(1):63. doi: 10.1212/WNL.0000000000002818 27306630

[20] Kelkar P. Letter Re: A Randomized Controlled Trial of Methotrexate for Patients With Generalized Myasthenia Gravis. Neurology (2017) 88(4):417. doi: 10.1212/WNL.0000000000003548 28115603

[21] Heckmann JM Bateman K. Letter Re: A Randomized Controlled Trial of Methotrexate for Patients With Generalized Myasthenia Gravis. Neurology (2017) 88(4):417. doi: 10.1212/WNL.0000000000003547 28115602

[22] Pasnoor M. Author Response: A Randomized Controlled Trial of Methotrexate for Patients With Generalized Myasthenia Gravis. Neurology (2017) 88(4):417–8. doi: 10.1212/WNL.0000000000003549 28115604

[23] Zinman L Ng E Bril V. IV Immunoglobulin in Patients With Myasthenia Gravis: A Randomized Controlled Trial. Neurology (2007) 68(11):837–41. doi: 10.1212/01.wnl.0000256698.69121.45 17353471

[24] Farmakidis C Pasnoor M Dimachkie MM Barohn RJ. Treatment of Myasthenia Gravis. Neurol Clin (2018) 36(2):311–37. doi: 10.1016/j.ncl.2018.01.011 PMC6690491 29655452

[25] Sharshar T Porcher R Demeret S Tranchant C Gueguen A Eymard B. Comparison of Corticosteroid Tapering Regimens in Myasthenia Gravis: A Randomized Clinical Trial. JAMA Neurol (2021) 78(4):426–33. doi: 10.1001/jamaneurol.2020.5407 PMC7871208 33555314

[26] Chinese Rheumatology Association. Chinese Guideline for the Diagnosis and Treatment of Rheumatoid Arthritis. Zhonghua Feng Shi Bing Xue Za Zhi (2010) 14:265–70. doi: 10.3760/cma.j.issn.1007-7480.2010.04.014

[27] Pasnoor M He J Herbelin L Dimachkie M Barohn RJ. Phase II Trial of Methotrexate in Myasthenia Gravis. Ann N Y Acad Sci (2012) 1275:23–8. doi: 10.1111/j.1749-6632.2012.06804.x PMC3564221 23278574

[28] Nair SC Jacobs JW Bakker MF Jahangier ZN Bijlsma JW van Laar JM. Determining the Lowest Optimally Effective Methotrexate Dose for Individual RA Patients Using Their Dose Response Relation in a Tight Control Treatment Approach. PLoS One (2016) 11(3):e148791. doi: 10.1371/journal.pone.0148791 PMC4795693 26987073

[29] Bergstra SA Allaart CF Stijnen T Landewe R. Meta-Regression of a Dose-Response Relationship of Methotrexate in Mono- and Combination Therapy in Disease-Modifying Antirheumatic Drug-Naive Early Rheumatoid Arthritis Patients. Arthritis Care Res (Hoboken) (2017) 69(10):1473–83. doi: 10.1002/acr.23164 27992656

[30] Burmester GR Kivitz AJ Kupper H Arulmani U Florentinus S Goss SL. Efficacy and Safety of Ascending Methotrexate Dose in Combination With Adalimumab: The Randomised CONCERTO Trial. Ann Rheum Dis (2015) 74(6):1037–44. doi: 10.1136/annrheumdis-2013- PMC4431334 24550168

