# Supplementary material for: A Randomized Open-Labeled Trial of Methotrexate as a Steroid-Sparing Agent for Patients With Generalized Myasthenia Gravis
Source: Front Immunol. 2022 Mar 18;13:839075. doi: 10.3389/fimmu.2022.839075 (PMC8971191; doi:10.3389/fimmu.2022.839075)
Supplement: Supplementary file 1 [file Table_1.docx]

**Supplement Table 1.** Prednisone daily dose taper monthly according to the following protocol.

| Daily Dose (mg) | Decrease Dose to (mg) |
| --- | --- |
| 80 | 70 |
| 70 | 60 |
| 60 | 50 |
| 50 | 40 |
| 40 | 30 |
| 30 | 25 |
| 25 | 20 |
| 20 | 15 |
| 15 | 10 |
| 10 | 7.5 |
| 7.5 | 5 |
| 5 | 2.5 |
| 2.5 | 0 |
